# Design of Ultra-Compact and Multifunctional Optical Logic Gate Based on Sb_2_Se_3_-SOI Hybrid Platform

**DOI:** 10.3390/nano14151317

**Published:** 2024-08-05

**Authors:** Liuni Yang, Qiang Liu, Haoyuan Liang, Minming Geng, Kejin Wei, Zhenrong Zhang

**Affiliations:** 1 Guangxi Key Laboratory of Multimedia Communications and Network Technology, School of Computer, Electronics and Information, Guangxi University, Nanning 530004, China; 2 Guangxi Key Laboratory for Relativistic Astrophysics, School of Physical Science and Technology, Guangxi University, Nanning 530004, China

**Keywords:** inverse design, Sb_2_Se_3_, direct binary search algorithm, reconfigurable optical logic gate, integrated optics

## Abstract

Optical logic devices are essential functional devices for achieving optical signal processing. In this study, we design an ultra-compact (4.92 × 2.52 μm^2^) reconfigurable optical logic gate by using inverse design method with DBS algorithm based on Sb_2_Se_3_-SOI integrated platform. By selecting different amorphous/crystalline distributions of Sb_2_Se_3_ via programmable electrical triggers, the designed structure can switch between OR, XOR, NOT or AND logic gate. This structure works well for all four logic functions in the wavelength range of 1540–1560 nm. Especially at the wavelength of 1550 nm, the Contrast Ratios for XOR, NOT and AND logic gate are 13.77 dB, 11.69 dB and 3.01 dB, respectively, indicating good logical judgment ability of the device. Our design is robust to a certain range of fabrication imperfections. Even if performance weakens due to deviations, improvements can be obtained by rearranging the configurations of Sb_2_Se_3_ without reproducing the whole device.

## 1. Introduction

Optical logic gates are among the most important basic units in optical signal processing, as well as in optical communication networks. A series of logic gates based on various schemes, such as photonic crystals [1,2,3,4,5,6], plasmonic waveguides [7], and semiconductor optical amplifier [8,9,10,11], have been proposed and proven. However, logic gates based on these schemes are large, function unitary and non-adjustable.

Integrated optical devices or systems are a hot research topic and the inverse design method has been extensively applied in the researches of nanophotonic devices recently. Many integrated optical devices, such as polarization beam splitter [12], waveguide bends [13,14], reflector [15], optical power splitters [16,17,18,19,20] and logic gates [21,22], were designed by using inverse design method and then prepared. Compared with devices constructed using traditional methods, structures invented using inverse design have higher degree of freedom and more compact layouts. For instance, Q. Lu et al. [21] used inverse design method to compose a NOT gate and an AND gate, the individual size of both is only 1.2 × 1.2 μm^2^. H. Qi et al. [22] designed an integrated photonic circuit with two all-optical switches controlling the input states of an all-optical XOR logic gate by using inverse design method and fabricated it out, the size of the whole circuit is 2.5 × 7 μm^2^. The fundamental of inverse design is to determine the target performance of the device, then adopt diverse optimization algorithms to calculate and design the required configuration by using computer without manual participation [23]. By using this method, high performance devices can be easily developed under certain constraints (e.g., specified footprints or structures) without manually tedious parameter adjustment works, saving massive resources. Inverse design method can be based on genetic algorithm [15], direct binary search (DBS) algorithm [12,16,17,19,20,24], or deep learning algorithm [25], etc. Among them, the DBS algorithm has received increasing attention due to its fast convergence speed and operation convenience. The optical devices mentioned in this paragraph have high integration, but their functions cannot be adjusted. In order to develop reconfigurable devices, phase change materials have been introduced into the device construction process using inverse design method.

Phase change materials have attracted widespread attention due to their multiple states, which enable the reconfiguration of optical devices. Usually, the phase change materials can be switched between two states: crystalline or amorphous. The conversion between these two states can be activated by external thermal, optical and electrical triggers. This conversion is reversible but non-volatile, once the state switches, there is no need to maintain continuous external triggering. The difference in refractive index between the amorphous and crystalline states of materials is significant, so that different functions can be achieved on the same device. So far, phase change materials have been used in creations of tunable optical devices like switches [26,27,28,29], power splitters [30,31], logic gates [32,33] and mode converters [34], etc. Especially, the complex refractive index at 1550 nm of phase change material Sb_2_Se_3_ is 3.285 + 0.000i for amorphous state and 4.050 + 0.000i for crystalline [35], the corresponding extinction coefficient is 0 for both states. This means that Sb_2_Se_3_ barely owns optical absorption at 1550 nm, indicating extremely low loss compared to the common phase-change materials Ge_2_Sb_2_Te_5_ (GST) [26,29] and Ge_2_Sb_2_Se_4_Te_1_ (GSST) [30]. There are a few photonic logic gates designed by using inverse design with DBS algorithm based on material Sb_2_Se_3_. For example, Z. Peng et al. [32] simulated a 2 × 2 μm^2^ optical logic gate, which could be used as an AND gate or a XOR gate when its Sb_2_Se_3_ part was switched between amorphous or crystalline. Y. Zhang et al. [33] designed two logic gates, one of which exhibited the function of OR gate and NOT gate, and the other implemented XOR gate and AND gate when the state of Sb_2_Se_3_ was changed. Sb_2_Se_3_ can be prepared using various methods such as pulse laser deposition [36,37,38], magnetron sputtering [39,40], and thermal evaporation [39,41,42]. In addition, the refractive index of Sb_2_Se_3_ in amorphous state is similar to that of silicon, which can be well integrated into standard silicon-on-insulator (SOI) integrated photonic platforms. Therefore, it can be considered to incorporate Sb_2_Se_3_ into the SOI platform to form hybrid structures for reconfigurable optical logic gates.

In this paper, we demonstrate an ultra-compact (4.92 μm × 2.52 μm for the optimal region) and multifunctional optical logic gate based on Sb_2_Se_3_-SOI hybrid platform by using inverse design method with DBS algorithm. Once the structure is prepared, by selecting appropriate amorphous and crystalline distribution of Sb_2_Se_3_ via external electrical triggers that are supplied by an application specific integrated circuit (ASIC) [30,43], the designed structure can switch between four types of logic gates that act as an OR gate, a XOR gate, a NOT gate or an AND gate. Unlike the optical logic gate design based on Sb_2_Se_3_ material introduced above, in our design, each Sb_2_Se_3_ rectangular deposited in the silicon layer is relatively independent and the corresponding amorphous/crystalline states can be controlled individually. This provides the possibility of achieving more device functions on the same structure. We also analyze the device robustness against manufacturing imperfections. The simulation results show that the device can still commendably perform the expected logic function within a certain fabrication deviation range. If the performance weakens due to manufacturing imperfections while the designed structure works as a logic XOR gate, a NOT gate, or an AND gate, improvement can be obtained by simply recalculating the distribution of Sb_2_Se_3_ amorphous/crystalline states. In other words, improvement can be easily achieved by changing the states of Sb_2_Se_3_ via external triggers without reproducing the whole device.

## 2. Inverse Design of the Multifunctional Optical Logic Gate

**Figure 1:**
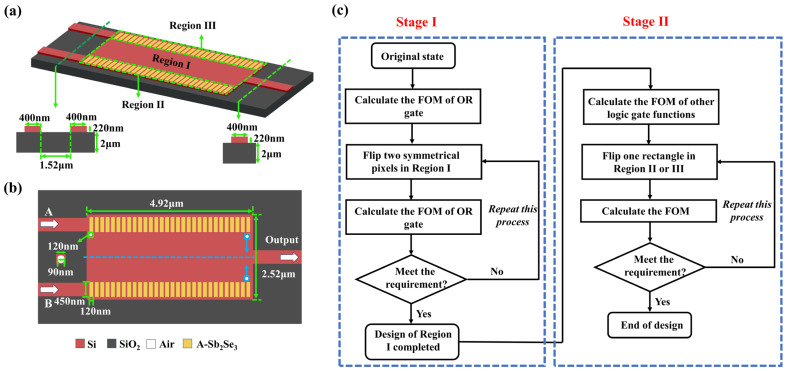
The basic structure and design process of the optical logic gate; (**a**,**b**) initial design layout and parameters, A-Sb_2_Se_3_ stands for amorphous Sb_2_Se_3_, the white arrow in (**b**) represents the direction of light transmission, the blue arrow represents selecting two symmetrical pixels simultaneously by column and the selecting direction order when designing Region I; (**c**) DBS algorithm optimization process of our designed logic gate.

Our integrated optical logic gate was constructed by using the inverse design method with DBS algorithm and air cylindrical lattice structure. As shown in Figure 1a,b, the material for design was set as a 220 nm processable silicon (marked in red) layer on a 2 µm silica substrate (marked in grey). The 4.92 × 2.52 μm^2^ design area was divided into three regions. The Region I was divided into 480 (40 × 12) square pixels with an individual size of 120 × 120 nm^2^. Each pixel was assigned one of the following two states according to calculation: unetched and etched, which was equivalent to fill the corresponding pixel center with an air cylinder (marked in white) with a radius of 45 nm and a thickness of 220 nm. Region II and Region III each contained a column of Sb_2_Se_3_ rectangles (marked in yellow) with 40 nm silicon gaps between them. The size of each rectangle was 450 × 120 nm^2^ and its thickness was 220 nm. Rather than placing Sb_2_Se_3_ thin film on the surface, we embedded the Sb_2_Se_3_ rectangles into the silicon layer to pursue stronger light field control ability [26,29,30,31,32,33,34]. According to Ref. [16], we added a 60 nm protection layer around each region to avoid edge being etched through. The width of input/output waveguides was set as 400 nm [29,32,33] and the gap between two input ports was set as 1.52 μm. All simulations in this research were carried out used commercial software Ansys Lumerical FDTD 2020 R2.4. The wavelength of laser input was set as 1550 nm and the launched mode was set as TE_0_ mode initially.

The DBS algorithm inverse design process for our designed logic gate is shown in Figure 1c. A critical step of inverse design was to set a figure-of-merit (*FOM*), which represented the approaching degree of the current configuration’s performance to the target function when the states of pixels in Region I and Sb_2_Se_3_ rectangles in Region II or III were changed. The *FOM* should approach a certain convergence value as the number of iterations increased to ensure the correctness of the device optimization. The entire optimization process is divided into two steps. In the Stage I, Region I is optimized separately to complete the logic OR gate design. In the Stage II, Region II and III are treated as a whole to complete the logic XOR gate, logic NOT gate and logic AND gate design, respectively. The original state of pixels in Region I was set as “unetched” and that for rectangles in Region II and III was set as “amorphous”. During the simulation, the state of each pixel was flipped to “etched” sequentially and the corresponding *FOM* was calculated at the same time. If the *FOM* was improved, the state of the calculating pixel would be retained, otherwise would return back to “unetched”. One iteration was completed when all pixels were traversed and the next round would start with the current device structure as the new initial structure. The iterative process would stop when the *FOM* no longer grew with the number of iterations, i.e., the *FOM* converged. A similar process was carried out on Region II then Region III to determine the crystalline/amorphous state of each Sb_2_Se_3_ rectangle. It is worth noting that when calculating the state of pixels in Region I, instead of selecting one pixel at a time, selecting two symmetrical pixels simultaneously for simulation obtained better performance devices and double computational efficiency. For XOR gate, NOT gate and AND gate, in order to obtain more possibilities during the simulation process, only one rectangle in Region II or III is flipped at a time. By changing the distribution of etched air holes and crystalline/amorphous Sb_2_Se_3_ rectangle, the effective refractive index of the device can be adjusted, thereby regulating the output of optical signals to achieve different logic gate functions. Our simulation results can be reproduced using the same parameters and following the same steps. The figures shown in this paper are optimal results obtained after multiple calculations, thus eliminating the influence of stochastic uncertainties.

Normally, ‘0’ is used to indicate no signal while ‘1’ means a signal input. As a logic gate, there are four types of input pair via Port A and Port B of our design, namely ‘00’, ‘01’, ‘10’ and ‘11’. Since we expect our design owns the function of an OR gate, a XOR gate, a NOT gate and an AND gate, and the output of any aforementioned logic gate is always ‘0’ when the input is ‘00’. Hence the ‘00’ input situation is not considered in subsequent simulations.

We first calculated the configuration (Region I only) of an OR gate according to the following *FOM*:

(1) $$FOM_{OR}=T_{01}+T_{10}+T_{11}$$

where *T*_01_, *T*_10_ and *T*_11_ represent the light transmittance detected at the output port when the input is ‘01’, ‘10’, or ‘11’, respectively. Based on the simulated configuration of OR gate, we further calculated the state of Sb_2_Se_3_ rectangles in Region II and III according to following *FOM* formulas to achieve the function of XOR gate, NOT gate or AND gate:

(2) $$FOM_{XOR}=1+T_{01}+T_{10}-T_{11}-\left|T_{01}-T_{10}\right|$$

(3) $$FOM_{NOT}=1+T_{01}-T_{11}$$

(4) $$FOM_{AND}=1-\left|T_{11}-0.7\right|-\left|T_{01}-0.35\right|-\left|T_{10}-0.35\right|$$

When the output should be judged as logic ‘1’, we expect a high transmittance and set it to a positive value, otherwise a negative value is set. The item $\left|T_{01}-T_{10}\right|$ is used to balance the error of the outputs when inputting ‘01’ and ‘10’ for the XOR gate. When simulating the AND gate, we set the judging threshold as 0.5, the target value as 0.7 for *T*_11_ and 0.35 for *T*_01_ and *T*_10_. Because the output corresponding to ‘11’ input should equal to the sum of transmittances corresponding to ‘01’ or ‘10’ input due to the symmetrical structure of Region I.

Contrast Ratio (*CR*) [44] is a criterion to evaluate the difference between logic ‘1’, and logic ‘0’. It is defined as:

(5) $$CR=10lg(T_{1}/T_{0})$$

where *T*_1_ stands for the minimum optical transmittance of device for logic ‘1’, and *T*_0_ means the maximum transmittance for logic ‘0’ [1]. The higher the *CR* value, the stronger the device’s ability to distinguish between logic ‘1’ and logic ‘0’.

## 3. Simulation Results

As described above, we designed an optical logic gate based on the Sb_2_Se_3_-SOI hybrid platform using the inverse design method with the DBS algorithm. The layout of Region I (SOI) in our design is fixed. By changing the state of Sb_2_Se_3_ (Region II and III) to crystalline or amorphous, functions of OR gate, XOR gate, NOT gate and AND gate can be obtained. The function details of the logic gate we designed are as follows.

### 3.1. OR Logic Gate

**Figure 2:**
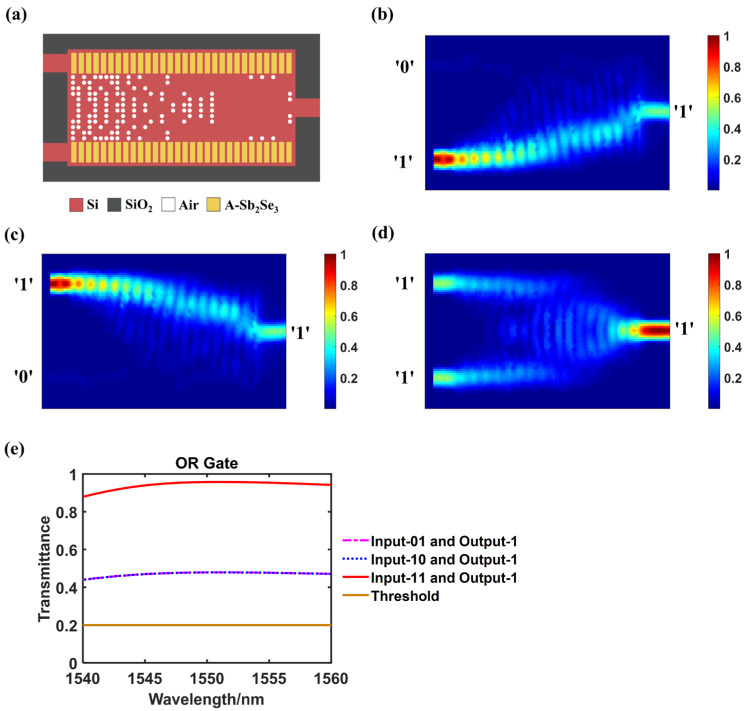
OR logic gate. (**a**) The configuration of the designed structure when act as an OR gate, A-Sb_2_Se_3_ stands for amorphous Sb_2_Se_3_; (**b**–**d**) the simulated light field intensity distributions when inputting ‘01’, ‘10’ or ‘11’ separately; (**e**) Transmittance variation curves of different output logic states within the wavelength range of 1540–1560 nm.

Figure 2a–d present the optimized layout and the simulated light field distribution when our designed structure act as an OR logic gate. As shown in Figure 2b–d, as long as there is input from either Port A or Port B, or both, there is an output that is higher than the logical judging threshold, achieving the function of an OR gate.

Figure 2e shows the transmittance changes of different output logic states in the wavelength range of 1540–1560 nm. Table 1 displays the normalized optical transmittances at 1550 nm wavelength and the binary output truth table when the device works as a logical OR gate. We set the logic judging threshold as 0.2. Transmittance larger than this value will be expressed as logic ‘1’, otherwise it will be expressed as logic ‘0’. The device can effectively distinguish between logic ‘1’ and ‘0’ based on the design threshold within the wavelength range of 1540–1560 nm. At 1550 nm wavelength, when the input is ‘00’ and there is no output, it is judged as logic ‘0’. When the input pairs are ‘01’, ‘10’ and ‘11’, the corresponding transmittances are 0.478, 0.478 and 0.957, respectively, which are all larger than the threshold then can be determined as logic ‘1’. Logic ‘1’ and ‘0’ can be clearly distinguished that the functionality of the logical OR gate is realized.

### 3.2. XOR Logic Gate

**Figure 3:**
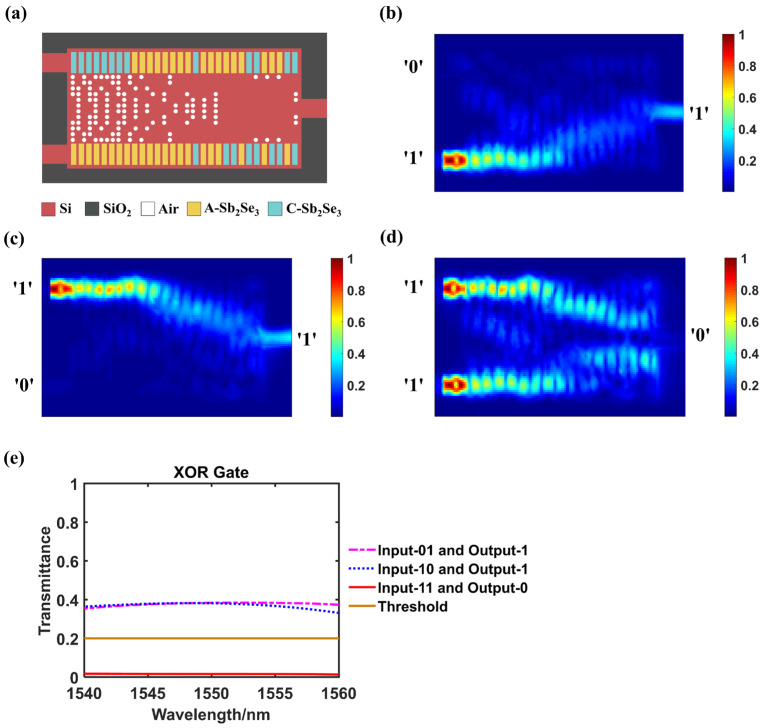
XOR logic gate. (**a**) The configuration of designed structure when act as a XOR gate, A-Sb_2_Se_3_ stands for amorphous Sb_2_Se_3_ and C-Sb_2_Se_3_ stands for crystalline Sb_2_Se_3_; (**b**–**d**) The simulated light field intensity distributions when inputting ‘01’, ‘10’ or ‘11’ separately; (**e**) Transmittance variation curves of different output logic states within the wavelength range of 1540–1560 nm.

When the truth values at the input ends are different, the XOR gate should output logic ‘1’, otherwise it should output logic ‘0’. Figure 3a–d show the optimized configuration and the simulated light field distributions of XOR logic gate. Figure 3e shows the transmittance changes of different output logic states in the wavelength range of 1540–1560 nm.

Accordingly, Table 2 lists the key data and truth table of XOR gate. The judging threshold is also set as 0.2. As shown in Figure 3b–e and Table 2, When the input pair are ‘01’ or ‘10’, the outputs are 0.383 and 0.382, which are higher than the threshold that could be determined as logic ‘1’. When the input is ‘11’, the transmittance is 0.016, which is too weak that can be judged as logic ‘0’. The judgement is valid in a wavelength range of 1540–1560 nm. The CR of photonic XOR logic gate is 13.77 dB, indicating a strong discriminative ability of this design.

### 3.3. NOT Logic Gate

**Figure 4:**
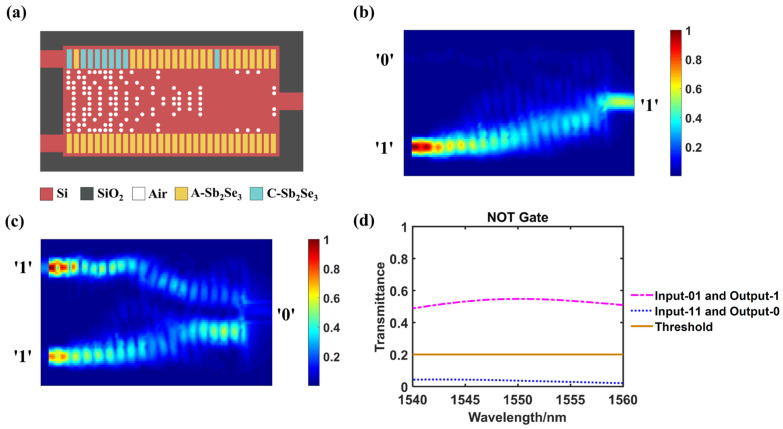
NOT logic gate. (**a**) The configuration of designed structure when act as a NOT gate, A-Sb_2_Se_3_ stands for amorphous Sb_2_Se_3_ and C-Sb_2_Se_3_ stands for crystalline Sb_2_Se_3_; (**b**,**c**) The simulated light field intensity distributions when inputting ‘0’ or ‘1’ at Port A, respectively, Port B is used for control; (**d**) Transmittance variation curves of different output logic states within the wavelength range of 1540–1560 nm.

Figure 4a–c present the optimized configuration and the simulated light field distributions of the NOT logic gate. We set Port B as a control waveguide with a constant light, so that there is still an output can be detected as logic ‘1’ when no input (logic ‘0’) from Port A (Figure 4b). While Port A has a signal input (logic ‘1’), there is almost no output that can be detected which is judged as logic ‘0’ (Figure 4c). The NOT gate determination function is valid in the wavelength range of 1540–1560 nm (Figure 4d).

The exact data of the NOT gate operating at 1550 nm wavelength are listed in Table 3. The judging threshold is also set as 0.2. When the input at Port A is ‘0’, the transmittance is 0.547, which is greater than the threshold that can be determined as logic ‘1’. While the input at port A is ‘1’, the transmittance is only 0.037 then is judged as logic ‘0’. The CR of logic NOT gate is 11.69 dB.

### 3.4. AND Logic Gate

**Figure 5:**
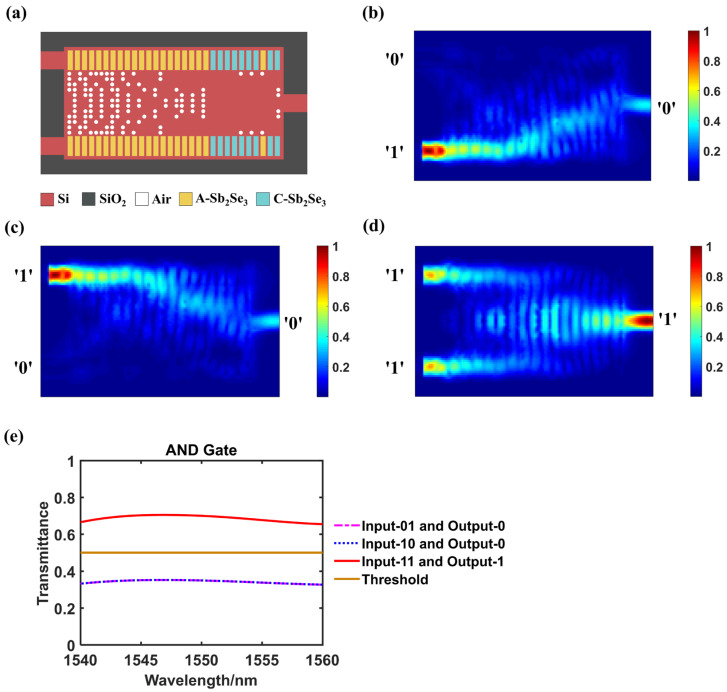
AND logic gate. (**a**) The configuration of designed structure when act as an AND gate, A-Sb_2_Se_3_ stands for amorphous Sb_2_Se_3_ and C-Sb_2_Se_3_ stands for crystalline Sb_2_Se_3_; (**b**–**d**) The simulated light field intensity distributions when inputting ‘01’, ‘10’ or ‘11’ separately; (**e**) Transmittance variation curves of different output logic states within the wavelength range of 1540–1560 nm.

Figure 5a–d show the layout as well as the light field intensity distribution at 1550 nm wavelength of AND logic gate, and Table 4 displays its detail data. The transmittance changes of different output logic states in the wavelength range of 1540–1560 nm is shown in Figure 5e. Slightly different from other logic gate functions, the judging threshold for AND gate is set as 0.5. Hence when inputting ‘01’ or ‘10’, the transmittances are 0.350 and 0.349, respectively, which are determined as logical ‘0’ since they are below the threshold. While the input is ‘11’, the transmittance is 0.700, which is larger than the threshold that is judged as logic ‘1’. The function of logical AND gate is implemented. The CR of AND gate is 3.01 dB.

In practice, when the input pair for logic gate is ‘11’, there may be inconsistencies in the amplitude or phase of the light launched at the two input ports. Therefore, we studied the impacts on outputs of different logic functions of our design when there is a ±5% deviation in the amplitude of two inputs. Outcomes caused by phase differences ranging from 0 to π between two inputs were also investigated. The simulation results showed that the impact of inconsistent amplitude or phase input on device performance is minimal.

### 3.5. Manufacturing Tolerance Analysis

**Figure 6:**
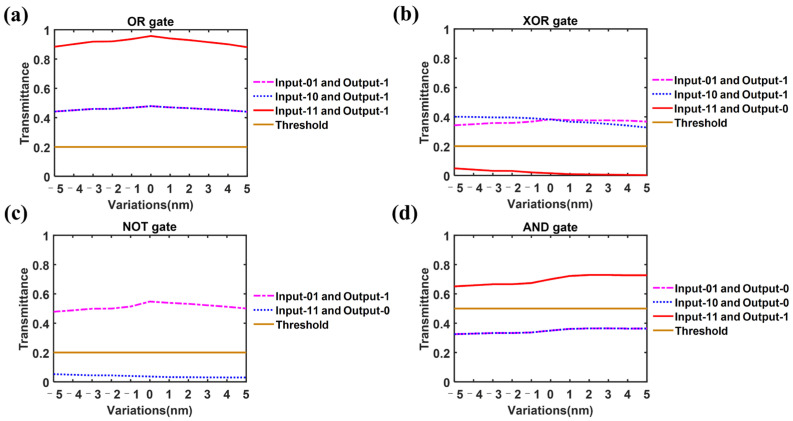
Simulated light transmittance at the output port under certain fabrication deviations. The error range for the diameter of the holes in Region I as well as the length and width of the rectangles in Region II or III is ±5 nm (abscissa). (**a**) output transmittance of logic OR gate; (**b**) output transmittance of logic XOR gate; (**c**) output transmittance of logic NOT gate; (**d**) output transmittance of logic AND gate.

Because of the unavoidable fabrication imperfections in practice, it is essential to discuss the effect of fabrication tolerance on the designed logic gate. We simulated the performance changes of the device under different bias conditions and display them in Figure 6. The error range for the diameter of the holes in Region I as well as the length and width of the rectangles in Region II or III is set to ±5 nm [45] (abscissa). As shown in Figure 6, the changes in transmittances of logic gate are not significant and all within the range that can be correctly determined. This suggests that even if there are offsets within the set range due to fabrication imperfections in practice, the designed logic gate can still complete the expected functions. It is worth noting that, in addition to simulating the case where the device manufacturing tolerance is a fixed value as described above, we also simulated the case where the manufacturing tolerance randomly varies obeyed a uniform statistical distribution within a ±5 nm range. Compared to devices with fixed ±5 nm manufacturing errors, devices with randomly distributed manufacturing tolerances have better performance, which proves strong device robustness.

**Figure 7:**
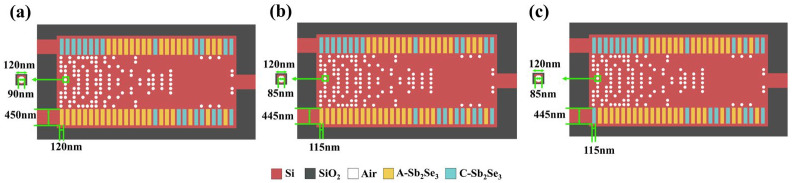
(**a**) The configuration of XOR gate designed with set ideal parameters; (**b**) the configuration of XOR gate with parameter bias; (**c**) the configuration of XOR gate recalculated with deviations. The states of Sb_2_Se_3_ rectangles in Region II and III are changed. A-Sb_2_Se_3_ stands for amorphous Sb_2_Se_3_ and C-Sb_2_Se_3_ stands for crystalline Sb_2_Se_3_.

We found that there is a relatively obvious change in the transmittance under some bias conditions. When the device operates as a logic XOR gate and the diameter of the holes in Region I as well as the length and width of the rectangles in Region II or III (Figure 6b) gradually decreases, the transmittance corresponding to ‘11’ input and logic ‘0’ output increases that may lead to a decrease in CR, i.e., a weakening in device’s performance. If such a situation occurs in actual preparation, in order to improve device performance, we can recalculate the state of Sb_2_Se_3_ rectangles (Regions II and III) using the actual fabrication parameters based on the current designed structure (Figure 7). New optimal distribution of the amorphous/crystalline states of Sb_2_Se_3_ rectangles can then be easily implemented using ASIC controlled by computer programming. On the other hand, the pattern of Region I, which needs to go through complex processes like laser engraving and etching during fabrication, requires no changes. As shown in Table 5, the CR of improved configuration is 11.26 dB.

Overall, the simulation results reveal that under certain fabrication imperfections, the device has good robustness and can still achieve the predetermined functions. Even if the device’s performance is weakened due to fabrication deviation, improvement can still be achieved by simply rearranging the state of phase change materials Sb_2_Se_3_, which is trigger by computer programming ASIC, without reprocess the entire device.

## 4. Conclusions

In this research, we design an ultra-compact, reconfigurable multifunctional optical logic gate by using inverse design method with DBS algorithm based on Sb_2_Se_3_-SOI integrated platform. The device’s footprint is only 4.92 × 2.52 μm^2^. By arranging different crystalline/amorphous distributions of Sb_2_Se_3_ rectangles via electrical heating provided by programmable ASIC, functions of OR, XOR, NOT and AND logic gate can be realized. The device works well in all four logic functions within the wavelength range of 1540–1560 nm. The CRs for XOR, NOT and AND logic gate function at 1550 nm wavelength are 13.77 dB, 11.69 dB and 3.01 dB, respectively, indicating good logical judgment ability. We also analyze the device robustness against fabrication imperfections. The preparation deviation range is specified as: ±5 nm for the diameter of etched holes in Region I and the side length of rectangles in Region II or III of Sb_2_Se_3_. Under this circumstance, our design still maintains excellent performance, indicating a good manufacturing tolerance. Moreover, if the device’s performance is weakened due to fabrication imperfections, improvements can be obtained by simply rearranging the distribution of Sb_2_Se_3_ amorphous/crystalline states according to actual parameters. In summary, changes in functionality and improvements in performance can be easily achieved by changing the states of Sb_2_Se_3_ via external triggers without reproducing the whole device.

## Figures and Tables

**Table 1:** The normalized input, transmittance and the truth table for OR logic gate.

Input A	Input B	Threshold	Optical Transmittance	Binary Output
0	0	0.2	0	0
0	1	0.2	0.478	1
1	0	0.2	0.478	1
1	1	0.2	0.957	1

**Table 2:** The normalized input, transmittance and the truth table for XOR logic gate.

Input A	Input B	Threshold	Optical Transmittance	Binary Output
0	0	0.2	0	0
0	1	0.2	0.383	1
1	0	0.2	0.382	1
1	1	0.2	0.016	0

**Table 3:** The normalized input, transmittance and the truth table for NOT logic gate.

Input A	Input B for Control	Threshold	Optical Transmittance	Binary Output
0	1	0.2	0.547	1
1	1	0.2	0.037	0

**Table 4:** The normalized input, transmittance and the truth table for AND logic gate.

Input A	Input B	Threshold	Optical Transmittance	Binary Output
0	0	0.5	0	0
0	1	0.5	0.350	0
1	0	0.5	0.349	0
1	1	0.5	0.700	1

**Table 5:** Comparison of CR for XOR gate calculated with parameter deviations before or after optimization.

Designed Structures	XOR Gate Designed with Set Parameters (Figure 7a)	XOR Gate with Parameter Bias (Figure 7b)	Optimized XOR Gate with Parameter Bias (Figure 7c)
Contrast Ratio (CR)	13.77 dB	8.43 dB	11.26 dB

## Data Availability

Data available in a publicly accessible repository.
